# Pyogenic Psoas Abscess in a Patient With Chronic Low Back Pain: A Case Report

**DOI:** 10.7759/cureus.50330

**Published:** 2023-12-11

**Authors:** Ohoud Almana, Khawla Ben Yahia, Adnan Aldhabab, Rawan Aljohani, Ahmad Bilal

**Affiliations:** 1 General Practice, Ibn Sina National College for Medical Studies, Jeddah, SAU; 2 General Practice, Alfaisal University, Riyadh, SAU; 3 General Practice, King Abdulaziz University, Jeddah, SAU; 4 General Surgery, Al-Dar Hospital, Medina, SAU

**Keywords:** computed tomography, percutaneous abscess drainage, diabetes mellitus, low back pain, psoas abscess

## Abstract

A psoas abscess, a rare and clinically significant entity, poses diagnostic challenges due to its nonspecific presentation and diverse etiologies. This case report explores a compelling instance of a 58-year-old male with type 2 diabetes mellitus and chronic low back pain diagnosed with a large pyogenic psoas abscess. The patient presented with worsening right-sided lower back pain, fever, and chills. Clinical examination revealed lumbar tenderness and limited spinal mobility. Laboratory results indicated elevated inflammatory markers. Computed tomography identified a large abscess involving the right iliopsoas. A working diagnosis of pyogenic psoas abscess was established, prompting surgical intervention. This case emphasizes the diagnostic complexity of a psoas abscess, particularly in individuals with predisposing factors. Successful management, involving advanced imaging, targeted antibiotics, and minimally invasive interventions, underscores the efficacy of a multidisciplinary approach. Heightened clinical suspicion, prompt diagnosis, and tailored interventions are crucial for navigating the complexities of this condition successfully.

## Introduction

A psoas abscess, a rare yet clinically significant entity, poses a diagnostic challenge due to its nonspecific presentation and potential association with various underlying etiologies [1]. While typically categorized as either primary, resulting from hematogenous spread, or secondary, arising from adjacent infectious foci or structural abnormalities, the condition necessitates a multidisciplinary approach for accurate diagnosis and optimal management. Notably, predisposing factors for a psoas abscess include immunocompromised states, chronic medical conditions, such as diabetes mellitus, and previous spinal interventions [2]. Pyogenic origins, particularly Staphylococcus aureus, predominate in primary cases, requiring judicious use of imaging modalities and microbiological analyses for precise identification [3]. The literature underscores the importance of prompt intervention, combining both medical and surgical strategies, to mitigate potential complications and ensure favorable patient outcomes. In this context, we present a compelling case of a 58-year-old male with type 2 diabetes mellitus and chronic low back pain who was diagnosed with a large pyogenic psoas abscess.

## Case presentation

A 58-year-old male, with a past medical history significant for type 2 diabetes mellitus and chronic low back pain, presented to our emergency department with a three-week history of progressively worsening right-sided lower back pain, fever, and chills. The patient reported no recent trauma or urinary symptoms. He denied any history of intravenous drug use or recent invasive procedures. Upon admission, the patient appeared ill, with a temperature of 38.5°C (101.3°F), heart rate of 110 beats per minute, and blood pressure of 130/80 mmHg.

The patient's physical examination revealed tenderness over the right lumbar paravertebral area, exacerbated by passive hip flexion on the right side. There was a limited range of motion in the lumbar spine with associated discomfort. Neurological examination demonstrated no focal deficits, and the abdominal examination was unremarkable. Laboratory investigations revealed an elevated white blood cell count of 14,500 cells/mm³ with a left shift. C-reactive protein and erythrocyte sedimentation rates were markedly elevated at 78 mg/L and 85 mm/hour, respectively (Table 1). Blood cultures were obtained, and the patient was started on broad-spectrum antibiotics pending culture results.

**Table 1:** Initial laboratory results

Laboratory Parameter	Result	Reference Range	Units
Hemoglobin	12.5	13.5 - 17.5	g/dL
White Blood Cell Count	14,500	4,000 - 11,000	cells/mm³
Platelet Count	300,000	150,000 - 450,000	cells/mm³
Neutrophil Count	10,500	2,500 - 7,500	cells/mm³
Lymphocyte Count	3,000	1,000 - 4,000	cells/mm³
Erythrocyte Sedimentation Rate	85	0 - 20	mm/hour
C-reactive Protein	78	0 - 5	mg/L
Serum Glucose	180	70 - 100	mg/dL
Blood Urea Nitrogen	20	8 - 20	mg/dL
Serum Creatinine	1.2	0.6 - 1.3	mg/dL
Alanine Aminotransferase	25	7 - 56	U/L
Aspartate Aminotransferase	30	5 - 40	U/L

**Figure 1:**
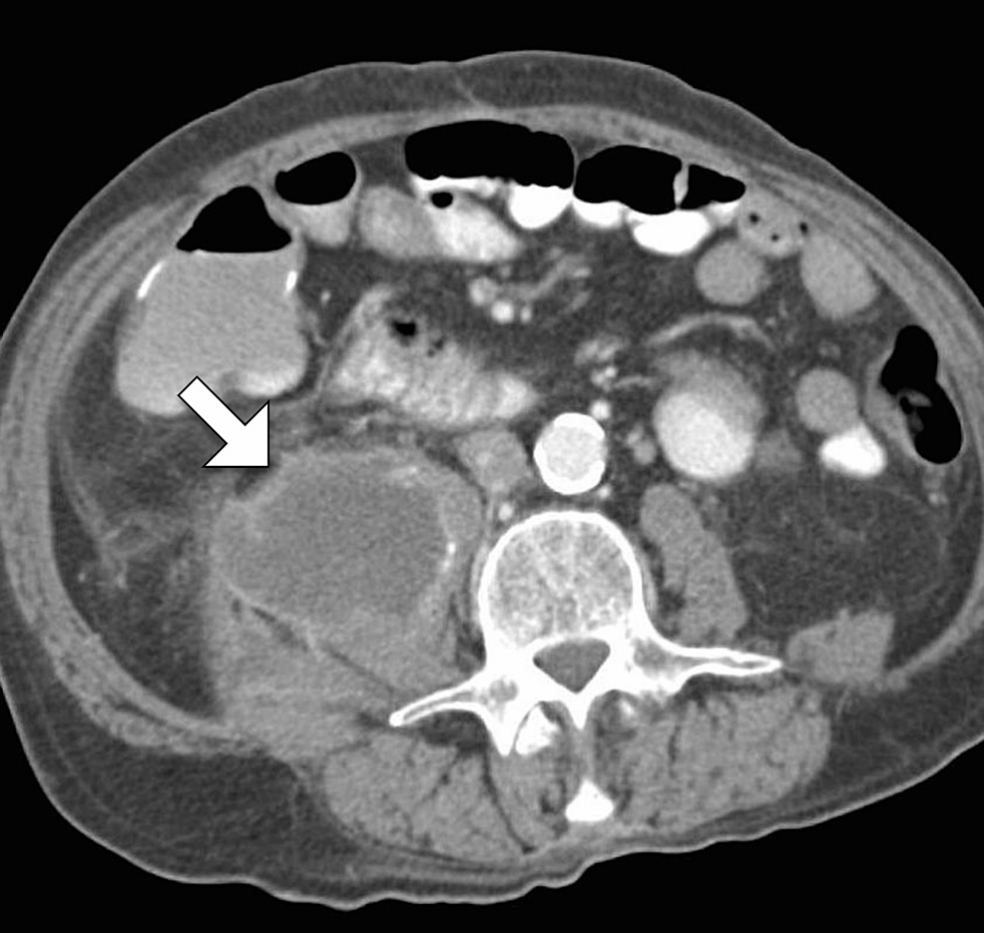
Axial CT image of the abdomen reveals a collection within the right psoas muscle (arrow) with a thickened enhancing wall, consistent with an abscess CT: computed tomography

**Figure 2:**
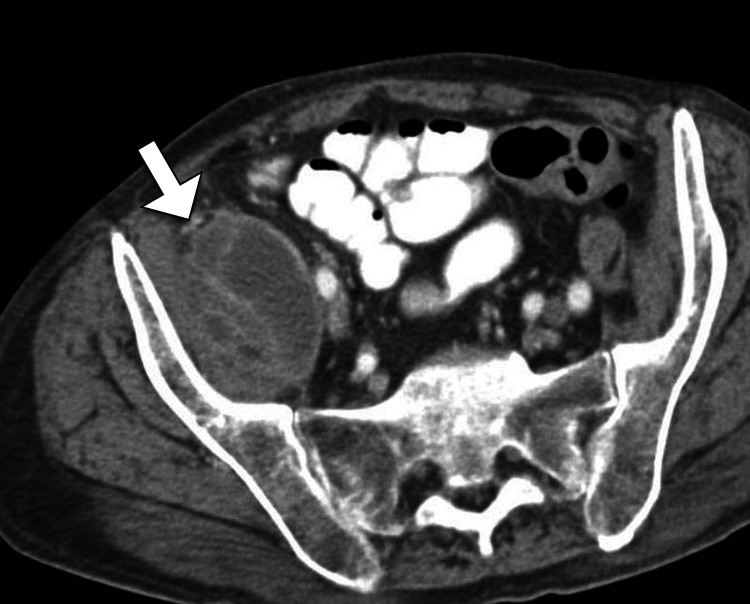
Axial CT image of the pelvis reveals a collection within the right psoas muscle (arrow) with a thickened enhancing wall, consistent with an abscess CT: computed tomography

Considering the clinical presentation, the differential diagnosis included infectious etiologies such as pyogenic or tuberculous spondylodiscitis, as well as non-infectious causes such as neoplastic processes. To further evaluate the patient's condition, a computed tomography scan of the abdomen was performed. The CT findings revealed a large volume collection with enhancing walls involving the entire right iliopsoas (Figures 1-2).

The working diagnosis was a pyogenic psoas abscess, given the combination of clinical presentation, imaging findings, and elevated inflammatory markers. The patient was promptly transferred to the surgical service for further management.

A percutaneous drainage procedure was performed under image guidance by the interventional radiology team, yielding purulent fluid consistent with a bacterial abscess. Cultures grew Staphylococcus aureus, prompting adjustment of antibiotic therapy to intravenous Vancomycin. The patient's clinical condition gradually improved over the next few days, with a resolution of fever and a downtrend in inflammatory markers.

The hospital course was complicated by the development of transient acute kidney injury related to antibiotic therapy, necessitating adjustment of drug dosages and close nephrology consultation. The patient completed a six-week course of intravenous antibiotics and was transitioned to oral antibiotics for an additional four weeks.

Follow-up at three months revealed complete clinical recovery with no residual symptoms. The patient's inflammatory markers had normalized, confirming the successful management of this challenging case of right psoas abscess.

## Discussion

The psoas muscle, a deep-seated structure in the lumbar region, is an uncommon site for abscess formation. Psoas abscesses may arise from hematogenous spread, contiguous infections, or direct extension from adjacent structures. Psoas abscesses can be categorized into primary and secondary, each with distinct etiologies [3]. Primary abscesses typically result from hematogenous spread, often associated with systemic infections, while secondary abscesses arise from adjacent infectious processes. The presented case underscores the significance of this entity in patients with predisposing factors such as diabetes mellitus and chronic low back pain [2].

Recognizing the protean clinical presentations of psoas abscesses is paramount for early diagnosis. Patients may exhibit nonspecific symptoms such as fever, flank pain, and constitutional signs. However, the challenge lies in differentiating these symptoms from other abdominal and musculoskeletal pathologies. The microbial landscape of the psoas abscess, often dominated by Staphylococcus aureus in primary cases, necessitates a judicious selection of antibiotics informed by culture results, as evidenced in our patient's successful outcome with targeted intravenous vancomycin therapy [1,4].

Recognition of predisposing factors of psoas abscess is crucial for risk assessment. Immunocompromised states, diabetes, and prior spinal interventions are notable risk factors [1]. The diagnostic pathway highlights the pivotal role of radiological investigations in confirming the diagnosis and delineating the extent of the abscess. Furthermore, the timely intervention through percutaneous drainage, guided by imaging, not only facilitated microbiological confirmation but also contributed to the overall therapeutic success [5]. The procedural aspect of this case prompts reflection on the evolving role of minimally invasive techniques in the management of psoas abscesses, emphasizing the importance of a collaborative effort involving interventional radiology and surgical expertise [4].

The challenges encountered during the hospital course, including transient acute kidney injury related to antibiotic therapy, underscore the need for vigilant monitoring and tailored adjustments in drug regimens. This complication, although managed successfully in our case, prompts consideration of the delicate balance required in optimizing therapeutic interventions in complex clinical scenarios.

## Conclusions

In conclusion, this case underscores the clinical importance of recognizing a psoas abscess as a diagnostic challenge, particularly in individuals with predisposing factors such as diabetes mellitus and chronic low back pain. The successful management of this case, using advanced imaging, targeted antibiotics, and minimally invasive interventions, highlights the success of a multidisciplinary approach in improving patient outcomes. Our experience highlights the need for heightened clinical suspicion, prompt diagnostic evaluation, and tailored therapeutic interventions to navigate the complexities of this condition successfully.
